# Case report: Successful treatment of chorioptic mange in two Belgian draft horse foals with topical ivermectin

**DOI:** 10.3389/fvets.2024.1427400

**Published:** 2024-11-20

**Authors:** Marieke Brys, Stien Den Hondt, Veronique Saey, Edwin Claerebout, Koen Chiers

**Affiliations:** ^1^Laboratory of Veterinary Pathology, Department of Pathobiology, Pharmacology and Zoological Medicine, Faculty of Veterinary Medicine, Ghent University, Merelbeke, Belgium; ^2^DAP Stien Den Hondt, Lier, Belgium; ^3^Laboratory of Parasitology, Faculty of Veterinary Medicine, Ghent University, Merelbeke, Belgium

**Keywords:** Belgian draft horse, *Chorioptes bovis*, ivermectin, treatment, mange

## Abstract

**Introduction:**

Various draft horse breeds, especially breeds with abundant feathering on the lower limbs, are known to be susceptible to chorioptic mange. Clinical signs of chorioptic mange encompass intense pruritus leading to self-mutilation and hair loss, thickening of the epidermis, and the formation of hyperkeratotic crusts and scabs. Despite the frequent occurrence and high impact of this condition, treatment options are limited, with a conspicuous absence of registered products formulated for equines, and especially foals. This limited availability of approved products highlights the necessity for alternative approaches to effectively address chorioptic mange in draft horse populations, given the severity of the clinical signs and their negative impact on the well-being of infested horses.

**Methods:**

Two 9-months old Belgian draft horse foals with clinical signs of severe pruritus and skin scaling on the distal legs were included. Both horses tested positive for living mites by means of superficial skin scrapings. Topical treatment with ivermectin at a dose of 1 mg/kg body weight was applied twice with 1 week interval, directly onto the distal legs of the horses.

**Results:**

In both cases, the mites were morphologically identified as *Chorioptes bovis*. Negative scrapings were obtained 7 days after the second treatment. Pruritus resolved in both horses within 2 days after the first treatment. Clinical signs consisting of skin scaling and crusting in the pastern region had resolved as well at 1 week and 3 weeks after the second treatment, respectively.

**Conclusion:**

This case report presents the first successful treatment of two clinical cases of chorioptic mange in Belgian draft horse foals with 1 mg/kg topical ivermectin, applied directly onto the distal legs.

## 1 Introduction

Chorioptic mange, caused by the mite *Chorioptes bovis*, is a parasitic dermatitis affecting both wild and domestic ungulates (1). While its prevalence is notably observed in cattle and small ruminants, its occurrence in equines, especially foals, remains relatively undocumented. Chorioptic mange in horses is characterized by pruritus, alopecia and the development of hyperkeratotic crusts and scales in the affected areas, typically localized to the lower limbs and in severe cases extending to the ventral abdomen (2, 3). This infestation often predisposes affected areas to secondary bacterial infections, amplifying clinical complexity. Currently, no specific products are tailored for the treatment of chorioptic mange in equines, and effective therapeutic management strategies often entail labor-intensive and time-consuming treatments including clipping of the feathering and multiple washings of the affected areas (3). A recent study explored the efficacy of topical moxidectin in treating chorioptic mange in adult draft horses (4). However, while these findings highlighted positive outcomes in adult horses, it is crucial to exercise caution when considering moxidectin treatment for foals due to potential toxicity risks associated with high doses (5, 6). Ivermectin, a broad-spectrum antiparasitic agent, has demonstrated efficacy against various mite species and is presumed to have a greater safety margin in young animals (7). However, its topical application in chorioptic mange cases in equines warrants further investigation. Through a detailed examination of two clinical cases, this report aims to elucidate the clinical manifestation, diagnosis, and successful treatment of chorioptic mange in two draft horse foals, emphasizing the efficacy of topical ivermectin as a therapeutic intervention.

## 2 Case description

In February 2024, two 9-month-old Belgian draft horse foals, one female and one male, were presented with severe pruritus, manifesting excessive biting, stomping and scratching their lower legs. This behavior was observed by the owner for several months. Both foals had been weaned at the age of 120 days. Before weaning, the individual foals were housed with their dam in separate paddocks. Following weaning and during the time of this study, the foals were housed together in an outside paddock with free access to an inside shelter. Beside severe pruritus, excessive scaling of the skin on all 4 lower legs was observed, especially in the pastern cavity. No other clinical signs were noted. However, the adult horses in the field adjacent to the paddock, including both dams of the foals, also displayed signs of extensive pruritus during the stable visits.

## 3 Diagnostic assessment, treatment and outcome

**Figure 1:**
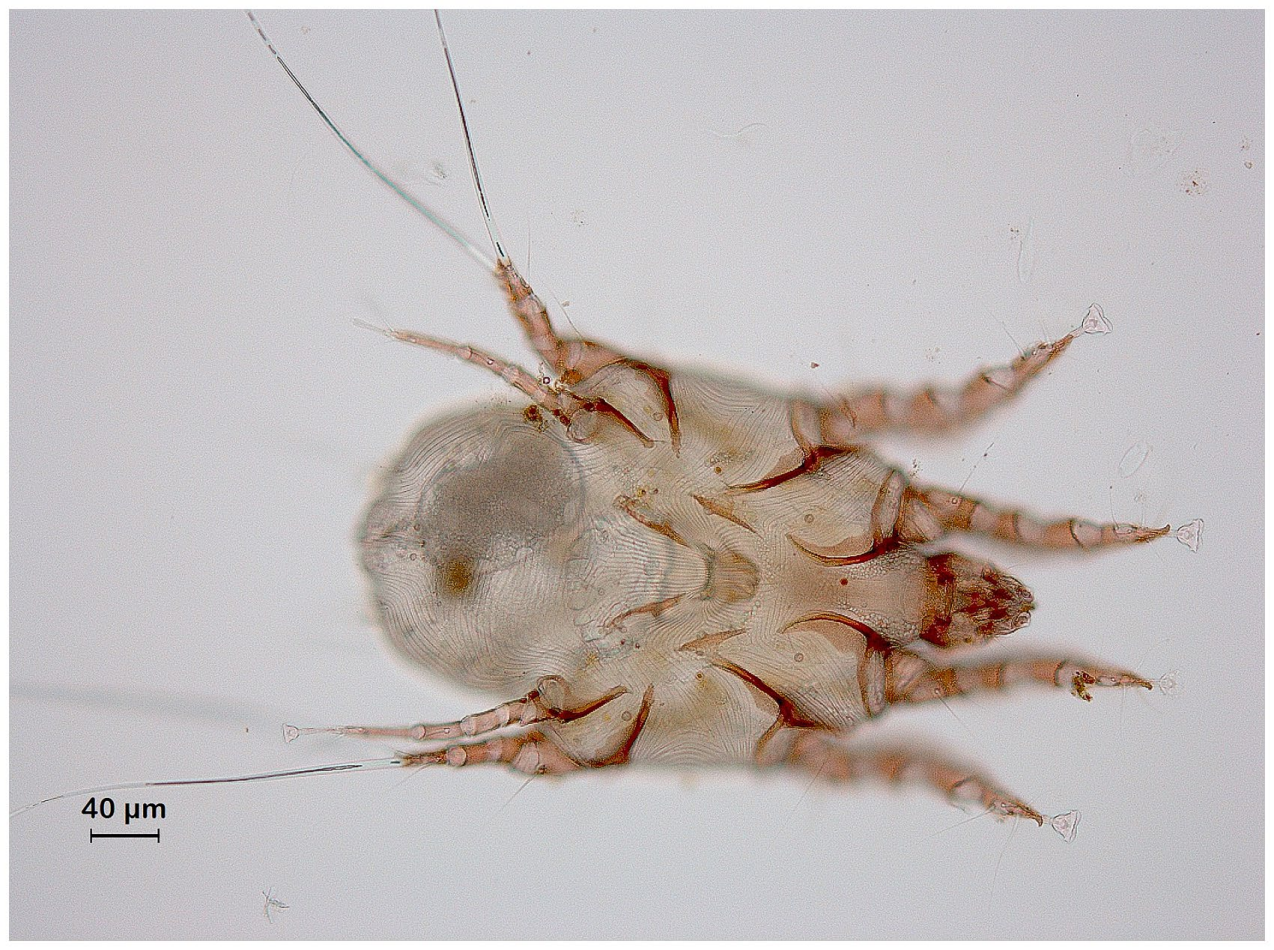
Adult stage of *Chorioptes bovis* in the skin scraping sample of the filly obtained before topical treatment with ivermectin.

**Figure 2:**
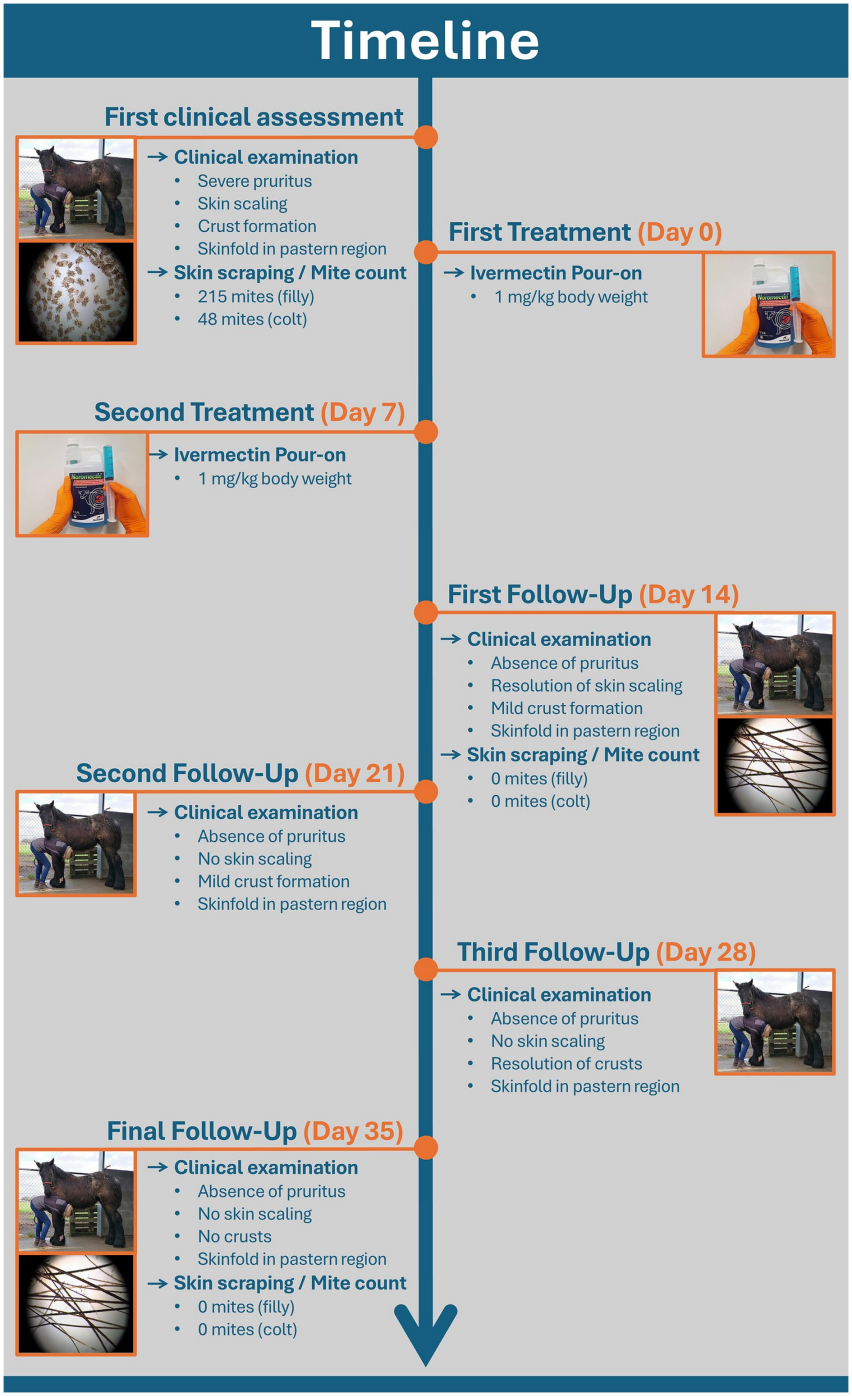
Schematic timeline displaying treatment, sampling intervals, clinical examinations and relevant clinical findings in both foals.

Since both foals exhibited distress upon palpation of the lower legs, the animals were sedated with 0.3 mL detomidine and 0.3 mL butorphanol IV. The feathering on the lower legs was not clipped, as requested by the owner. First clinical inspection of both foals revealed agitation due to pruritus and the presence of scaling of the skin on the distal limbs. In both horses, there was moderate crusting and distinct skin thickening in the pastern region, along with the presence of a horizontal skinfold. The prominence of this skinfold was mainly observed in the filly, while the colt only exhibited moderate skin thickening in this region. These lesions were only discernible upon thorough palpation due to the extensive feathering on the lower limbs, characteristic of the Belgian draft horse breed. After sedation and palpation, superficial skin scrapings of the distal legs were obtained for direct examination under a stereo microscope (Euromex DZ.5040). Samples were examined within 24 h after collection. Scrapings were obtained in both horses from the left front leg over an area of 3 × 3 cm by use of a surgical blade. Direct examination of these samples revealed the presence of living mites, identified as *Chorioptes bovis* through the morphological criteria established by Sweatman (1) (Figure 1). No other mite species were detected. Mite counts amounted to 215 and 48 in the filly and colt, respectively. Upon diagnosis of chorioptic mange, it was decided to administer treatment consisting of 1 mg/kg ivermectin pour-on (Noromectin pour-on 5 mg/mL, Norbrook). The foals’ weight was estimated at 300 kg and 60 mL Ivermectin pour-on (15 mL for each leg) was distributed over the skin between the hairs on the lower legs from the coronary band up to the level of the carpus/tarsus. Treatment was repeated after 7 days, regarding the life cycle of mange mites. Due to training on handling, no sedation was needed to facilitate the clinical examination during the second treatment. Seven days after the second treatment, additional skin scrapings were obtained and subsequently examined for the presence of living mites, as previously described. Follow up examinations consisted of weekly visual inspections up to 4 weeks after the second treatment, when a final collection of skin scraping samples was performed. Within 2 days after the first treatment, pruritus had resolved completely. One week after the second treatment, scaling of the skin had resolved as well. At this time point, skin scrapings from both foals were negative for the presence of living mites. Crusts in the pastern region had cleared at 3 weeks after the second treatment. Both visual inspection during weekly follow up examinations and information from the owner confirmed the continual absence of pruritus over this period of time. During the final sampling, 4 weeks after the second treatment, still no living mites were present in the skin scrapings of both foals and skin lesions remained absent. Figure 2 displays the schematic timeline of this case, including treatment, sampling intervals, clinical examinations and relevant clinical findings in both foals. Because both dams of the foals also displayed extensive pruritus during every stable visit, it was decided to sample both animals as well by means of superficial skin scrapings for the presence of mites. Analyses of these samples also revealed an infestation with *C. bovis* mites in both cases as cause of pruritus.

## 4 Discussion

*Chorioptes bovis* is suspected to be highly prevalent in draft horses with abundant feathering on the lower limbs (8–11). The transmission routes include direct transmission or transmission through contaminated fomites and bedding (12). In addition, these mites can survive in the environment for up to 69 days (13). A notable aspect of this case is that both dams of the foals were also found to be infested with *C. bovis*. These mares could therefore represent a potential source of mite infestation for the foals through direct contact before weaning or through contamination of the environment (e.g., bedding, fomites). No environmental treatment was implemented in this case and possible reinfestation of the foals may occur even without direct contact with the infested adult horses. Therefore, regular follow-up is warranted.

Clinical signs of mange include intense pruritus, scaling and crusting of the skin, alopecia, and the development of multiple wounds on the lower limbs. Alopecia and wound formation are primarily attributed to self-mutilation resulting from the intense pruritus. Mange has also been associated with the onset or complication of chronic progressive lymphedema (CPL) in Belgian draft horses (14). The latter is characterized by hallmark lesions, including skinfolds and nodules, which primarily manifest in the pastern region starting from the age of 3 years old, and subsequently advance to the proximal regions of the lower limbs as the condition worsens and the horse ages (15). Particularly in the filly foal, a horizontal fold in the pastern cavity was observed. This observation therefore may correspond to the first stage lesions of CPL, although it is unknown whether this lesion is a direct result of mite infestation or represents indeed an early onset of CPL.

The young age of the foals in which this mite infestation was detected, is also remarkable. To our knowledge, there have been no reports of infestations with *C. bovis* in such young horses. This young age also limits and complicates possible treatment options. Adult draft horses can be successfully and safely treated with pour-on moxidectin (1.5 mg/kg body weight), but this high dose of moxidectin could potentially have a neurotoxic effect in young horses with lower body condition scores (6). Ivermectin has a broader safety margin in young animals, and a pour-on dose of 1 mg/kg has been found to be safe in warmblood foals (16).

In the interest of resistance management, it should be noted that pour-on products, in addition to their efficacy against ectoparasites, also have an effect on gastrointestinal nematodes, although the latter may be diminished due to reduced transcutaneous absorption. In a study carried out by Gökbulut et al. (17) it was established that a high fecal egg count reduction of gastrointestinal nematodes could be obtained through application of pour-on ivermectin at 5 times the recommended oral dose of 0.2 mg/kg body weight, or the 1 mg/kg as used in both foals in this case.

It was decided not to clip the lower limbs, since this was specifically requested by the owner. However, our results indicate that there was still sufficient efficacy of topical ivermectin, when applied on the skin in between the hairs of the lower limbs. This method is more labor-intensive, as care must be taken to apply the product directly onto the skin and not the hair coat. Clipping of the lower limbs should be recommended to ensure adequate absorption of topical products and to visualize lesions appropriately.

To the best of our knowledge, we present in this paper the first report of the successful treatment of chorioptic mange in equines/foals with pour-on ivermectin.

## 5 Conclusion

This case report presents the successful treatment of two clinical cases of chorioptic mange in Belgian draft horse foals with 1 mg/kg body weight pour-on ivermectin, applied directly onto the distal legs.

## Data Availability

The original contributions presented in the study are included in the article/supplementary material, further inquiries can be directed to the corresponding author.
